# Risk of Respiratory Infectious Diseases and the Role of Methylphenidate in Children with Attention-Deficit/Hyperactivity Disorder: A Population-Based Cohort Study

**DOI:** 10.3390/ijerph18115824

**Published:** 2021-05-28

**Authors:** Dian-Jeng Li, Yi-Lung Chen, Ray C. Hsiao, Hsiu-Lin Chen, Cheng-Fang Yen

**Affiliations:** 1 Department of Addiction Science, Kaohsiung Municipal Kai-Syuan Psychiatric Hospital, Kaohsiung 80276, Taiwan; u108800004@kmu.edu.tw; 2 Department of Nursing, Meiho University, Pingtung 91200, Taiwan; 3 Graduate Institute of Medicine and School of Medicine, College of Medicine, Kaohsiung Medical University, Kaohsiung 80708, Taiwan; 4 Department of Psychology, Asia University, Taichung 41354, Taiwan; elong@asia.edu.tw; 5 Department of Healthcare Administration, Asia University, Taichung 41354, Taiwan; 6 Department of Psychiatry and Behavioral Sciences, University of Washington School of Medicine, Seattle, WA 98105, USA; rhsiao@u.washington.edu; 7 Department of Psychiatry, Children’s Hospital and Regional Medical Center, Seattle, WA 98105, USA; 8 Department of Respiratory Therapy, College of Medicine, Kaohsiung Medical University, Kaohsiung 80708, Taiwan; 9 Division of Neonatology, Department of Pediatrics, Kaohsiung Medical University Hospital, Kaohsiung Medical University, Kaohsiung 80708, Taiwan; 10 Department of Psychiatry, Kaohsiung Medical University Hospital, Kaohsiung Medical University, Kaohsiung 80708, Taiwan

**Keywords:** attention-deficit/hyperactivity disorder, respiratory infection, methylphenidate

## Abstract

Children with attention-deficit/hyperactivity disorder (ADHD) are commonly affected by medical illness. The aim of the present study was to explore the risks of contracting respiratory infectious diseases (RIDs), including upper and lower RIDs and influenza, in children with ADHD. We also examined whether methylphenidate has a protective effect regarding the risk of contracting RIDs among children with ADHD who have a history of methylphenidate treatment. Children in the Taiwan Maternal and Child Health Database from 2004 to 2016 were included in the present study. Upper and lower RIDs, influenza, ADHD, age, sex, and records of methylphenidate prescription were identified. A Cox proportional hazards regression model was used to estimate the significance of the risk of RIDs among children with ADHD in comparison with that among children without ADHD after adjustment for sex and age. The self-controlled case series analysis was conducted to examine the protective effect of methylphenidate treatment against RIDs. In total, 85,853 children with ADHD and 1,458,750 children without ADHD were included in the study. After controlling for sociodemographic variables, we observed that children with ADHD had significantly higher risks of upper RIDs, lower RIDs, and influenza infection than did those without ADHD. Among the children with ADHD who had a history of methylphenidate treatment, the risk of contracting RIDs was lower during the methylphenidate treatment period than during the nontreatment period. Children with ADHD had a higher RID risk than those without ADHD. Methylphenidate might reduce the risk of RIDs among children with ADHD who have a history of methylphenidate treatment.

## 1. Introduction

### 1.1. Association between Attention-Deficit/Hyperactivity Disorder and Medical Comorbidities

Attention-deficit/hyperactivity disorder (ADHD) is the most prevalent neurodevelopmental disorder and manifests as hyperactivity, inattentiveness, and impulsivity [1,2]. ADHD affects about 3.4% to 5.9% of the school-age population [1,3]. The lifetime prevalence of ADHD within children in Taiwan was reported to be up to 10.1% [4]. ADHD is associated with multidimensional disability, such as worsened quality of life [5] and impaired academic performance [6]. Individuals with ADHD are also at greater risk of various mental health–related comorbidities, such as suicidality [7], depression [8], and substance abuse [9]. In additional to mental disorders, ADHD is also often comorbid with medical illnesses. It is well known that the core ADHD symptoms increase the risks of accidents such as accidental burn injury [10] and injury-related mortality [11] among individuals with ADHD. However, research has demonstrated that medical illnesses in individuals with ADHD are widespread across different physical categories, such as cardiovascular, immunological, neurological, and gastroenterological diseases [12,13]. Although the relationship between ADHD and medical comorbidities is not fully understood, it highlights an urgent requirement for multidisciplinary medical services for children with ADHD [14].

### 1.2. ADHD, Methylphenidate, and Respiratory Infectious Diseases

Research has shown that children with ADHD have predominantly higher rates of allergic sensitization and allergic rhinitis than the general population, indicating the shared etiology of ADHD and immunological diseases [15,16]. However, the association between ADHD and respiratory infectious diseases (RIDs) has seldom been investigated. At the time of the coronavirus disease 2019 (COVID-19) outbreak, a U.S. population-based study reported a significantly elevated risk of COVID-19 infection and mortality among individuals with mental disorders, including ADHD [17]. Another study in Israel also demonstrated that the risk of contracting COVID-19 was higher in untreated individuals with ADHD than in those without ADHD [18]. Parental management, including keep children away from people with a cold, teaching children to wash their hands often, and reminding children not to touch their eyes, nose, and mouth is an important strategy for prevention of RIDs in children. However, all core symptoms of ADHD may increase the difficulty for children with ADHD to obey these rules and increase the opportunity for pathogen transmission [18]. The evidence of the association between ADHD and COVID-19 prompted our interest in the risk of RIDs among children with ADHD. RIDs, including upper and lower RIDs and influenza, are reported to be the leading cause of disability and mortality among children [19,20]. Examining the relationship between ADHD and RIDs in children can provide evidence for developing preventive strategies.

Methylphenidate is the most commonly used stimulant that is effective for reducing not only the severity of ADHD symptoms and ADHD-related dysfunction [21,22] but also the risks of accidental trauma [23], suicidality [24], and sexually transmitted infections [25] among individuals with ADHD. Whether methylphenidate can reduce the risk of RIDs in children with ADHD is undetermined. Given that methylphenidate is effective at improving inattention and impulsivity, it is reasonable to hypothesize that methylphenidate can increase ADHD children’s adherence to the hygiene requirements and reduce the risk of contracting RIDs. However, very few studies have examined the hypothesis. A recent study reported that the risk for COVID-19-positive was higher in untreated-ADHD subjects compared to non-ADHD subjects, while no higher risk was detected in treated ones [18]. Whether methylphenidate has a protective effect against contracting RIDs in children with ADHD warrants investigation.

### 1.3. Aim of the Current Study

The first aim of this population-based study was to investigate the risks of contracting upper RIDs, lower RIDs, and influenza among children with ADHD in comparison with those without ADHD. We hypothesized that compared with children without ADHD, children with ADHD would be more likely to contract upper and lower RIDs and influenza. The second aim of the study was to examine the effect of methylphenidate on reducing the risk of contracting RIDs among children with ADHD who received methylphenidate treatment. We hypothesized that for the children with ADHD who had received methylphenidate treatment, the risk of contracting RIDs would be significantly lower during the treatment period than during the nontreatment period.

## 2. Methods

### 2.1. Population

The present study included liveborn children with information regarding gestational age at birth in the Taiwan Maternal and Child Health Database (TMCHD), which covered 99.78% of births [26], from 2004 to 2016. The inclusion criterion of this study was that all children did not have missing information on their identity in the TMCHD. The exclusion criterion was if children were less than 5 years old in 2016, because ADHD diagnosis is possible from ages 5 years onward, and every participant had a minimum follow-up period of 1 year (to 2017). The current study was approved by the Research Ethics Committee of the China Medical University Hospital (approval number CMUH108-REC1-142).

### 2.2. Measure

#### 2.2.1. Exposure

In the current study, the primary exposure was the diagnosis of ADHD. Children were identified as having a diagnosis of ADHD if they received more than three outpatient diagnoses of ADHD (International Classification of Diseases, Ninth Revision, Clinical Modification [ICD-9-CM] code 314 and ICD-10 code F90) or one inpatient diagnosis of ADHD during the study period (from 1 January 2004, to 31 December 2017),

#### 2.2.2. Methylphenidate Treatment

For children with ADHD, we further examined whether they received methylphenidate treatment after ADHD diagnosis according to dispensed prescriptions recorded in the prescribed drug register with the Anatomical Therapeutic Chemical classification of methylphenidate (N06BA04).

#### 2.2.3. Outcome and Covariates

For all of the children in the TMCHD, the main outcomes included upper RIDs, lower RIDs, and influenza according to ICD-10 and ICD-9 in the Supplementary Table S1. The patients were followed up from 1 January 2004, to the primary outcome event, death, or the end of the study. The covariates in the current study were sociodemographic characteristics, namely age at the end of the follow-up period and sex.

### 2.3. Statistical Analyses

For the descriptive statistics, continuous variables and categorical variables were recorded as the mean with standard deviation and frequency with proportion, respectively. To estimate the difference between children with and without ADHD, the Pearson’s χ^2^ test was applied to compare categorical variables and an independent t test was used to compare continuous variables. To determine the risk of incident RIDs in children with and without ADHD, the Cox proportional hazards regression model was applied, with adjustment for age and sex. Hazard ratios (HRs) with 95% confidence intervals (CIs) were used to verify the risk and statistical significance of differences in incidence rates of upper RIDs, lower RIDs, and influenza. Children without ADHD served as the reference. An HR value greater than 1 indicated an increased risk of RIDs in children with ADHD compared with those without ADHD. If the 95% CI of the HR included the null value of 1, then no significant difference existed in the risk of RIDs between the groups.

To determine the beneficial effect of methylphenidate on RIDs, we performed a self-controlled case series (SCCS) analysis for within-individual comparisons. For individuals with ADHD, we divided the entire follow-up period into the nontreatment period and treatment period from the first date of ADHD diagnosis to the end of the research period (i.e., 31 December 2017), and the RIDs were treated as a recurrent event in the SCCS analysis. On the basis of previous studies [27,28], the treatment period was defined as the period when a sequence of methylphenidate prescriptions for ADHD were made with less than 6 months (183 days) between two consecutive prescriptions. For example, if the period between two treatments was less than 183 days, the begin of the treatment period was the date of the initial prescription, and the end of the treatment period was the date of the begin of next prescription. If participants only received one methylphenidate prescription throughout the follow-up period, then they were considered not to receive methylphenidate treatment. Because each patient served as his or her own control in the SCCS analysis, adjustments were automatically made for time-invariant factors (e.g., sex). A conditional Poisson regression model was used in the SCCS analysis, and the results are presented as relative risks (RRs) with 95% CIs. An RR value less than 1 suggested the protective effect of methylphenidate against RIDs in children with ADHD.

## 3. Results

### 3.1. Descriptive Analysis

In total, 85,853 children with ADHD and 1,458,750 children without ADHD were included in the study. The characteristics of demographic variables in children with and without ADHD are presented in Table 1. The age range of our participants was between 6 and 13 years, and the mean ages of the children with ADHD and those without ADHD were 10.1 ± 2.1 years and 9.7 ± 2.3 years, respectively. The proportion of boys among the children with ADHD was significantly higher than that among the children without ADHD (77.5% vs. 50.7%). Compared with children without ADHD, those with ADHD had significantly higher proportions of all types of RIDs, including upper RIDs (99.9% vs. 99.3%), lower RIDs (96.2% vs. 93.4%), and influenza (29.2% vs. 26.4%).

### 3.2. Risk of RIDs in Children with ADHD

As indicated in Table 2, the Cox regression model revealed that children with ADHD had significantly higher risks of upper RIDs, lower RIDs, and influenza than those without ADHD. After age and sex were controlled for, the risks for RIDs ranged from 1.07 to 1.17 (*p* < 0.001), with the highest risk noted for influenza. Protective effect of methylphenidate against RIDs in children with ADHD. Based on the SCCS analysis with a conditional Poisson regression model, we discovered that the children with ADHD had lower average risks for upper RIDs (RR, 0.47; 95% CI, 0.40–0.55), as well as for lower RIDs (RR, 0.36; 95% CI, 0.31–0.42), influenza (RR, 0.82; 95% CI, 0.77–0.87), and all types of RIDs (RR, 0.64; 95% CI, 0.61–0.68) during the methylphenidate treatment period compared with during the nontreatment period (Table 3).

## 4. Discussion

To the best of our knowledge, the present study initially reports the incidence of RIDs and effects of methylphenidate among children with ADHD. Although other research has explored the association between ADHD and the risk of COVID-19 infection [18], the present study further extends the knowledge of this association to various types of RIDs. The findings of the present study demonstrate that children with ADHD have higher risks of RIDs than those without ADHD. It is comparable to a previous study using nationwide claims data in Germany, reporting that children with ADHD had significant higher incidence of viral pneumonia than those without ADHD [29]. Our results show that regular treatment with methylphenidate could reduce the impact of ADHD on the risk of RIDs, indicating the protective effect of methylphenidate.

### 4.1. Possible Etiologies of the Increased Risk for RIDs

The significant association between ADHD and RIDs has several possible mechanisms. Initially, immunological problems might mediate the association between ADHD and RIDs. As is well known, the rates of allergic sensitization and allergic rhinitis are increased among children with ADHD [15,16]. Allergic inflammation in the airway impairs both the antiviral response and its epithelial barrier, and it is mediated by inflammatory cytokines, such as immunoglobulin E, interferon-α, and the α-chain of the high-affinity IgE receptor [30]. During allergic inflammation, upregulation of intracellular adhesion molecule 1 (ICAM-1) is observed [31]; given that ICAM-1 is the principal receptor for rhinovirus [32], the tissue affinity for rhinovirus infection increases. However, interleukin-13 is also secreted during allergic inflammation in the airway, which affects the ciliary beat frequency and enhances the viral invasion of nasal mucosa [33]. Consequently, allergic reaction may predispose patients with ADHD to RIDs.

In addition to immunological factors, the core symptoms of ADHD, such as inattention, hyperactivity, and impulsivity, interfere with the individual’s recognition of the need to comply with behaviors necessary for protecting against RIDs. For instance, inattention and impulsivity in children with ADHD may increase their likelihood of forgetting to wear a mask or to maintain social distancing during RID outbreaks. Moreover, research has found that parent–child conflict is common among children with ADHD and their parents [34]. The parents of children with ADHD may encounter difficulties in persuading their children to adopt the necessary measures for preventing RIDs. Further study is needed to examine the effects of ADHD symptoms and parent–child conflict on the risk of RIDs in children with ADHD.

### 4.2. Effect of Methylphenidate

Methylphenidate had been identified its protective effect on a reduction of different types of unintentional injuries in children and adolescents with ADHD, which is contributed by reducing symptoms of ADHD [28]. It is supposed that such protective effect of can be applied to other physical illness, which may be associated with symptoms of ADHD. The findings of the present study also demonstrate that methylphenidate helps in reducing the risk of RIDs among children with ADHD. The effect of methylphenidate in ameliorating the core symptoms of ADHD has been well established [35,36]. Notably, methylphenidate treatment in children with ADHD can significantly ameliorate teacher-reported ADHD symptoms in children with ADHD [35]. As is well known about the poor oral hygiene among children with ADHD [37], it is supposed that symptoms of inattention and hyperactivity may result in poor self-hygiene and low adherence to policies of infection control, such as keeping personal hygiene and wearing a mask. The potentially protective effect of methylphenidate may result from its effect on reducing symptoms of inattention and impulsivity and further on improving adherence for infection control. Moreover, mitigating the symptoms of ADHD in school is particularly crucial because schools, which constitute a semi-enclosed setting, are where RIDs can spread rapidly among children. A systemic review indicated that infectious illnesses spread quickly in educational settings such as schools and that hand hygiene interventions are effective for reducing the incidence of RIDs among schoolchildren [38]. For example, many countries have implemented school closures as part of physical distancing policies to slow the transmission of COVID-19 among children [39]. Moreover, a recent study identifies ADHD to be a risk factor for infection with COVID-19, while medications in treating with ADHD ameliorates this effect [18]. The characteristics of ADHD, such as inattention and hyperactivity, interfere with the ability to comply with the policies for prevention of COVID-19 infection [18]. Given the above evidence of benefits in reducing the risks of RIDs, the other study did not support this efficacy. For instance, a randomized multicenter clinical trial revealed that adult patients with ADHD who took methylphenidate had higher rates of acute tonsillitis and influenza infection than those taking placebo [40]. In sum, the current study demonstrated that methylphenidate may be potentially beneficial in reducing the risk of contracting RIDs in children with ADHD. Whether the protective effect of methylphenidate derive from its effect on ameliorating ADHD symptoms and increasing adherence to hygiene requirements warrants further examination.

On the other hand, some of the potentially harmful effects in methylphenidate should be noticed. By increasing catecholaminergic transmission, methylphenidate has their neurobiochemical relationship to the potential of abuse, misuse or intentional overdose [41]. Furthermore, several animal studies have demonstrated that methylphenidate increased oxidative stress and impaired function of the antioxidant system by an excessive level of reactive oxygen species, resulting in the DNA and cell death [42,43]. Therefore, alternative and non-pharmacological intervention in treating with symptoms of ADHD is also crucial in reducing risk of RIDs. Behavioral interventions, diet interventions, cognitive training and neurofeedback were also demonstrated their efficacy on ADHD core symptoms [22]. When prescribing methylphenidate with children with ADHD, clinicians should be aware of the undesirable effects and consider the indications of non-pharmacological interventions.

### 4.3. Limitations

There are several limitations of the present study. First, as a retrospective cohort study using the TMCHD, the interpretation of outcomes may have been limited by missing records for clinical variables. Furthermore, because the TMCHD only has the records of children born from 2004 and 2016, and they were followed by 2017, the age range of children in this study was restricted to 6 to 13 years, resulting in limited generalization to members of the pediatric population in other age ranges (e.g., 14 to 18 years). Second, the TMCHD is a database including naturalistic and clinical information, and possible confounding factors could not be controlled for to the same extent as they can be in clinical trials. For instance, prescription of methylphenidate is not randomized to groups of comparison. However, naturalistic studies can include representative individuals, enabling the results to be more reflective of real situations. Third, medication records were based on prescription data rather than actual consumption, resulting in potential misclassification because of common imperfect medication adherence. However, this may have had little impact on our results. Because of the limited drug regulations for methylphenidate and the young age of the children in this study, it is less likely that children who were not prescribed methylphenidate actually consumed it. Instead, it is more likely that children who were prescribed the medication were not adherent to it. In this case, the misclassified effect period should actually be attributed to the non-effect period, which is a bias in favor of nonmedication, and we observed a significant protective effect of methylphenidate against RIDs; thus, the true effect of methylphenidate should be more profound.

## 5. Conclusions

The present study reveals that children with ADHD have increased risks of contracting upper RIDs, lower RIDs, and influenza compared with those without ADHD. Regular treatment with methylphenidate is beneficial for reducing the risk of RIDs among children with ADHD. Establishing adequate interventions for infection control among children with ADHD is crucial, such as hygiene education, proper behavioral modification, and adjustments to school environments for social distancing purposes. Moreover, parents can adopt specialized parenting strategies to reduce the defiant and disruptive behaviors of children with ADHD, as recommended by the European ADHD Guidelines Group for preventing the spread of COVID-19 [44]. The results of the present study confirm the importance of regular treatment with methylphenidate in reducing the risk of RIDs among children with ADHD. Proper education of children and caregivers regarding the importance of medical adherence is especially critical during RID outbreaks.

## Figures and Tables

**Table 1:** Demographics and respiratory infections of children and adolescents with and without attention-deficit/hyperactivity disorder.

Variable	All (n = 1,544,603)	ADHD (n = 85,853)	Non-ADHD (n = 1,458,750)	*p*-Value
	Mean (SD)	Mean (SD)	Mean (SD)	
Age (years)	9.7 (2.3)	10.1 (2.1)	9.7 (2.3)	<0.001
	n (%)	n (%)	n (%)	
Sex				
Female	738,237 (47.8)	19,341 (22.5)	718,896 (49.3)	<0.001
Male	806,366 (52.2)	66,512 (77.5)	739,854 (50.7)	
Methylphenidate	-	43,158 (50.3)	-	
Outcome				
Upper respiratory infection	1,534,680 (99.4)	85,767 (99.9)	1,448,913 (99.3)	<0.001
Lower respiratory infection	1,445,540 (93.6)	82,612 (96.2)	1,362,928 (93.4)	<0.001
Influenza	409,624 (26.5)	25,106 (29.2)	384,518 (26.4)	<0.001
All respiratory infection	1,536,150 (99. 5)	85,779 (99.9)	1,450,371 (99.4)	<0.001

**Table 2:** Risks of respiratory infection in children with versus without attention-deficit/hyperactivity disorder.

Variable	Adjusted HR (95% CI)
Upper respiratory infection	1.07 (1.06–1.08)
Lower respiratory infection	1.09 (1.08–1.10)
Influenza	1.17 (1.16–1.19)
All respiratory infection	1.07 (1.06–1.08)

CI, confidence interval; HR, hazard ratio. Adjusted HR: Sex and age were controlled for.

**Table 3:** Self-controlled case series analysis of the risks of respiratory infection on the basis of methylphenidate treatment in children with attention-deficit/hyperactivity disorder.

Variable	Medication		Non-Medication		Adjusted RR (95% CI)
	Event	Cumulative Person-Time	Event	Cumulative Person-Time	
Upper respiratory infection	313	22,141,079	1100	41,320,667	0.47 (0.40–0.55)
Lower respiratory infection	331	22,141,079	1412	41,320,667	0.36 (0.31–0.42)
Influenza	2876	22,141,079	6102	41,320,667	0.82 (0.77–0.87)
All respiratory infection	3397	22,141,079	8235	41,320,667	0.64 (0.61–0.68)

RR, relative risk. Adjusted analysis: Time-invariant covariates (e.g., sex) were controlled for.

## Data Availability

The data presented in this study are available on request from the corresponding author.

## Supplementary Materials

The following are available online at https://www.mdpi.com/article/10.3390/ijerph18115824/s1, Table S1: Reference ICD9/10 code of all respiratory infection.
