# Supplementary material for: Risk of Respiratory Infectious Diseases and the Role of Methylphenidate in Children with Attention-Deficit/Hyperactivity Disorder: A Population-Based Cohort Study
Source: Int J Environ Res Public Health. 2021 May 28;18(11):5824. doi: 10.3390/ijerph18115824 (PMC8199289; doi:10.3390/ijerph18115824)
Supplement: Supplementary file 1 [file ijerph-18-05824-s001.zip › ijerph-1214630-supplementary.pdf]

Supplementary

Table S1. Reference ICD9/10 code of all respiratory infection.

icd-10			icd-9		
**Upper Respiratory Infection**	**Lower Respiratory Infection**	**Influenza**	**Upper Respiratory Infection**	**Lower Respiratory Infection**	**Influenza**
460	466	487	J00	J20	J00
461	480	488	J01	J21	J01
462	481		J02	J22	J02
463	482		J03		J03
464	483		J04		J04
465	484		J05		J05
	485		J06		J06
	486				J09
	490				J10
					J11
					J12
					J13
					J14
					J15
					J16
					J17
					J18
					J20
					J21
					J22
